# Supplementary material for: Predicting biomass of resident kōkopu (Galaxias) populations using local habitat characteristics
Source: PLoS One. 2023 Mar 14;18(3):e0261993. doi: 10.1371/journal.pone.0261993 (PMC10013890; doi:10.1371/journal.pone.0261993)
Supplement: S1 Table — (DOCX) [file pone.0261993.s001.docx]

**S1 Table. Summary statistics of the effects of each kōkopu species’ biomass on the biomass of other kōkopu within the same size class, after accounting for habitat features.**

| **Response** | **Predictor** | **Treatment DF** | **Residual DF** | **F** | **P** |
| --- | --- | --- | --- | --- | --- |
| Large kōkopu | | | | | |
| BK_LG_ | GK_LG_ | 1 | 55.72 | 0.07 | 0.792 |
| BK_LG_ | SJ_LG_ | 1 | 52.68 | 0.50 | 0.481 |
| GK_LG_ | BK_LG_ | 1 | 56.41 | 0.19 | 0.667 |
| GK_LG_ | SJ_LG_ | 1 | 48.93 | 0.30 | 0.584 |
| SJ_LG_ | BK_LG_ | 1 | 47.01 | 0.79 | 0.380 |
| SJ_LG_ | GK_LG_ | 1 | 51.12 | 1.53 | 0.222 |

‘BK’ is banded kōkopu, ‘GK’ is giant kōkopu, ‘SJ’ is shortjaw kōkopu, and ‘LG’ is large size class.
