# Supplementary material for: Predicting biomass of resident kōkopu (Galaxias) populations using local habitat characteristics
Source: PLoS One. 2023 Mar 14;18(3):e0261993. doi: 10.1371/journal.pone.0261993 (PMC10013890; doi:10.1371/journal.pone.0261993)
Supplement: S2 Table — (DOCX) [file pone.0261993.s002.docx]

**S2 Table. Instances of specific co-occurrence between banded (BK), giant (GK), and shortjaw (SJ) kōkopu small (SM) and large (LG) size classes observed across 57 sampled reaches.**

| **Kōkopu presence** | **Co-occurrence** |
| --- | --- |
| ALL | 1 |
| BK_LG_ | 1 |
| BK_LG_+BK_SM_ | 23 |
| BK_LG_+BK_SM_+GK_LG_ | 3 |
| BK_LG_+BK_SM_+GK_LG_+GK_SM_+SJ_SM_ | 1 |
| BK_LG_+BK_SM_+GK_SM_ | 6 |
| BK_LG_+BK_SM_+SJ_LG_ | 1 |
| BK_LG_+BK_SM_+SJ_LG_+SJ_SM_ | 1 |
| BK_LG_+BK_SM_+SJ_SM_ | 3 |
| BK_LG_+BK_SM_+SJ_SM_+SJ_LG_ | 2 |
| BK_LG_+BK_SM_+SJ_SM_+SJ_LG_+GK_SM_ | 1 |
| BK_LG_+GK_LG_ | 1 |
| BK_LG_+GK_LG_+BK_SM_+GK_SM_ | 3 |
| BK_LG_+GK_LG_+BK_SM_+GK_SM_+SJ_LG_ | 1 |
| BK_LG_+GK_LG_+GK_SM_+SJ_SM_ | 1 |
| BK_LG_+GK_LG_+SJ_LG_ | 1 |
| BK_LG_+GK_SM_+GK_LG_ | 2 |
| BK_LG_+SJ_LG_+BK_SM_+GK_SM_ | 1 |
| BK_SM_ | 1 |
| BK_SM_+GK_LG_+BK_LG_ | 1 |
| BK_SM_+GK_LG_+SJ_LG_ | 1 |
| GK_LG_+SJ_LG_ | 1 |
