# Supplementary material for: Predicting biomass of resident kōkopu (Galaxias) populations using local habitat characteristics
Source: PLoS One. 2023 Mar 14;18(3):e0261993. doi: 10.1371/journal.pone.0261993 (PMC10013890; doi:10.1371/journal.pone.0261993)
Supplement: S3 Table — (DOCX) [file pone.0261993.s003.docx]

**S3 Table. Instances of general co-occurrence between banded (BK), giant (GK), and shortjaw (SJ) kōkopu small (SM) and large (LG) size classes observed across 57 sampled reaches.**

|  | BK_LG_ | GK_LG_ | SJ_LG_ | BK_SM_ | GK_SM_ | SJ_SM_ |
| --- | --- | --- | --- | --- | --- | --- |
| BK_LG_ |  | 15 | 9 | 48 | 17 | 10 |
| GK_LG_ |  |  | 5 | 11 | 9 | 3 |
| SJ_LG_ |  |  |  | 9 | 4 | 5 |
| BK_SM_ |  |  |  |  | 14 | 9 |
| GK_SM_ |  |  |  |  |  | 4 |
| SJ_SM_ |  |  |  |  |  |  |
