# Supplementary material for: Predicting biomass of resident kōkopu (Galaxias) populations using local habitat characteristics
Source: PLoS One. 2023 Mar 14;18(3):e0261993. doi: 10.1371/journal.pone.0261993 (PMC10013890; doi:10.1371/journal.pone.0261993)
Supplement: Attachment — Submitted filename: Response to Reviewers.docx [file pone.0261993.s004.docx]

**Response to Reviewers**
*Please note that the line numbers in responses refer to the clean manuscript copy.*

**Editor**:

A major concern raised by both reviewers was that species identity was not included in the models. Although there is a large overlap in microhabitat it is still plausible that the different species have different ecological requirements. Both reviewers have given suggestions on how this can be dealt with. Another concern regards the representativeness of your results both to other systems and rivers entailing kökopu populations but also to other freshwater species. In your response letter you need to include point by point how you dealt with the reviewers' comments.

When submitting your revision, we need you to address these additional requirements:

Please ensure that your manuscript meets PLOS ONE's style requirements, including those for file naming. The PLOS ONE style templates can be found at 
<https://journals.plos.org/plosone/s/file?id=wjVg/PLOSOne_formatting_sample_main_body.pdf> and 
<https://journals.plos.org/plosone/s/file?id=ba62/PLOSOne_formatting_sample_title_authors_affiliations.pdf>

- This has been corrected.

We note that the grant information you provided in the ‘Funding Information’ and ‘Financial Disclosure’ sections do not match. When you resubmit, please ensure that you provide the correct grant numbers for the awards you received for your study in the ‘Funding Information’ section.

- This has been corrected.

We note that you have stated that you will provide repository information for your data at acceptance. Should your manuscript be accepted for publication, we will hold it until you provide the relevant accession numbers or DOIs necessary to access your data. If you wish to make changes to your Data Availability statement, please describe these changes in your cover letter and we will update your Data Availability statement to reflect the information you provide.

- We have created and linked a Figshare folder that includes the dataset.
  link: <https://figshare.com/s/686b54046be999b5ed95>
  DOI: 10.6084/m9.figshare.17203961

Please include a caption for figure 1

- This was given in-text. It is located between L305-311.

**Reviewer #1:**

The manuscript “Predicting biomass of resident kōkopu (Galaxias) populations using local habitat composition” evaluates the relationship among biomass of three endemic species of Galaxias and five instream habitat characteristics in New Zealand streams. I think the study is very well conducted and well written, and it is of great contribution to the field. This research is not only important for conservation of the species under study, but can also be used as a model to understand what drives variation in stream fish populations elsewhere. In general, I have mostly minor comments to the manuscript. My main concern is about the not inclusion of species identity in the models; all the three species are regarded as a single population in the models, which may be an issue because one may expect at least little differences in microhabitat selection. I think that adding the species identity as a random variable in the models will make the study more compelling and convincing to the readers.

I would suggest to use “local habitat characteristics' ' instead “composition”, as composition is more related to the content (like for a chemical compound or for a fish assemblage).

- L1: Swapped out “composition” for “characteristics”.

Is it a riverine or stream fish? Or a lake? Maybe state the type of freshwater habitat.

- L31: Described that stream reaches were being sampled.

L60-74: Regarding this, you may want to check Dala-Corte & De Fries (2018) study, which investigates very similar questions.

- Dala-Corte & De Fries (2018) was a great resource and its findings have been addressed between L70-73. The discussion has also benefitted from its themes.

L66. Give an example for what you mean with “same local environment”.

Have added “(eg., the same stream reach)” (L74).

L119: “habitat characteristics”. Review it elsewhere in the text.

Have fixed this issue throughout.

L119: State again which are these 3 species.

L126: Added species names.

L119-131: This paragraph is almost entirely a methods paragraph, I would move to methods.

This paragraph has been moved to methods (L192-197).

L133: I recommend “kokopu fish populations of three species endemic to New Zealand streams”.

Sentence has been changed, but similar detail has been added (L126).

L133: There is no need to state the use of AIC here, leave it to methods only. AIC is a widely known statistical approach.

Removed mentioning of AIC.

L136-142: Congratulations for the predictions.

Thank you, I am glad we now have even cooler ones to show.

L173-193: Were the individuals identified to species-level during the field?

Each fish was identified to species level (added L186).

L195: I think the author should include the species identity in the models (at least as a random variable). I know that the three species may largely overlap their microhabitat selection, readers will still think that it is very plausible that the different species have slightly different ecological requirements and behaviors. For instance, see Dala-Corte and De Fries (2018).

- Analyses now focus on each species separately. The biomass of other kōkopu species of the equivalent size-class not being examined have been added as random variables so we can account for species interactions and obtain a better representation of each species’ habitat use (L220-223). The new addition of variance components (in Table 1) indicates how much random variation the other species account for.

Table 1. Tables should not have these horizontal lines in the middle.

Lines were removed and replaced with shading.

L246: Like you did with large kokopu, start the small kokopu paragraph stating the results for the AIC models.

Results paragraph structuring is now more consistent between size-classes and mentions AIC information first.

L256-258: Actually, looking at the deltaAICc (smaller than 2), you do not have strong support to say that the model 1S is better than the 4S. So theoretically you should say that the best model is the 4S, which is the most simple. Yet, it is important to discuss the model 1S too.

This exact case is no longer included, but a similar case has occurred with the small banded kōkopu models. Although BK_S2_ was a simpler model, BK_S1_ was selected because it had a notably higher AICwt, indicating it was more likely to be the most parsimonious model. This is explained between L315-319.

Since you may be interested in the effect size of all the explanatory variables, you may want to inform the relative importance of the variables using the Summed Akaike Weights (SW) of all the models. See Burnham and Anderson (2002) and Giam and Olden (2016) if you have interest.

This was a great addition and is now shown in Table 3.

You may want to expand your comparisons to other studies with stream fish besides those with kokopu and trouts. For example, see Dala-Corte and De Fries (2018).

Have now included studies using other freshwater fish in citations throughout and in written examples (e.g., L371-374 & L405-408).

L283-292: You may want to recall the readers who are the kokopu species you studied.

Species have been added (L347).

L347. Ok, it was not locally important, but give an emphasis in this conclusion that upstream forest cover may be essential and probably explain your results.

L412-430: Paragraph has been changed due to different results. Paragraph now discusses and concludes that the relationships between local forest cover measurements and the biomass of large kōkopu was likely driven by food availably and interspecific competition.

L357-359: Ok, so benthic insects may be an important feeding resource. Make it clearer.

L440-443: Sentence has been made clearer.

L348-365. Indeed, I think that local riparian forest may be more important in driving refuge for small fish than in predicting the local input of terrestrial invertebrates, because upstream riparian forest may provide plenty of drifting terrestrial invertebrates to the pools.

L431-450: We believe refuge and aquatic prey are more important drivers of greater small kōkopu biomass because terrestrial prey are likely dominated by larger fish in the pools.

L367-373. You may want to take into consideration that the negative relationship between total bank cover and small kokopu biomass may just reflect that large kokopus displace the small ones from sites with more bank cover. In other words, an increase in bank cover may not be a conflict of interests for habitat restoration between large and small kokopus. If streams had no bank cover, you could probably don’t see any kokopu at all. To assess this one must sampling long stream reaches with few or no bank cover at all.

Acknowledged that our models describe the realised niche of each species’ size-class between L479-483. This is also emphasised in the paragraphs discussing the relationships between small kōkopu biomass and pool habitats (L378-380) and refuge use (L408-411).

**Reviewer #2:**

Dear Authors,

In my view, the basic premise of this manuscript – the need for better understanding and characterisation of ontogenetic variations in physical habitat preferences – is sound. However, I feel that there are a few aspects of this study that need to be tackled more substantively before it is ready for publication.

Conceptually, I see the value in pursuing the development of predictive models of fish biomass to support species management. As such, I see this as a valuable endpoint for this study. However, to strengthen the credibility and hence uptake of such models, in my view there needs to be greater transparency around the underlying data and assumptions that have been made. Furthermore, there is a need for validation and consideration of the transferability of the resulting models.

The study offers a valuable dataset on kōkopu habitat utilisation from multiple rivers on the west coast of the South Island of New Zealand. It is predicated on an assumption of potential habitat separation between ‘small’ and ‘large’ individuals arising from competitive displacement of smaller individuals from preferred habitats as indicated by previous studies. Below I make a few observations regarding the study methodology and some of the assumptions that I feel need addressing within the manuscript.

Firstly, it is indicated that the fish surveys took place during May and June (L147). This coincides with the spawning season of these species, yet the potential implications of this in terms of fish behaviour and habitat choice (and hence representativeness/transferability of these data and models) are not addressed in the manuscript. Mature individuals are known to aggregate prior to spawning and can undertake spawning migrations. Consequently, how representative are these observations (particularly for the ‘large’ group) of ‘normal’ habitat use and hence how transferable are the models?

- Addressed between L141-144. We believe the representativeness of our findings are not influenced by kōkopu spawning because kōkopu in our study were not notably fecund (examined using light stripping action) and because spawning occurs on the banks adjacent to adult habitats.

Secondly, observations for the three kōkopu species have been pooled, based on an assumption that they regularly co-exist and have similar habitat requirements (L121). Broadly speaking, yes, they all typically display an association with pool habitats and some degree of overlap in distribution does occur. However, I would suggest that there’s sufficient evidence to indicate a degree of spatial separation in the distribution of the three species (particularly beyond the South Island west coast rivers) broadly correlating with distance inland and elevation (giants lower gradient closer to the sea, bandeds low-mid gradients and further inland, shortjaws mid-higher gradients further inland still). I would also think there is some potential for differentiation in micro-habitat use between the species. Consequently, I feel there is a need to account for this potential in the analyses or at least demonstrate the assumption is sound. One of the benefits of the more modern mixed models as you have used here is that it would be simple to include species as a predictor variable and check the validity of this assumption.

- Analyses now focus on each species separately. The biomass of other kōkopu species of the equivalent size-class not being examined have been added as random variables so we can account for species interactions and obtain a better representation of each species’ habitat use (L220-223). The new addition of variance components (in Table 1) indicates how much random variation the other species account for.
- As now mentioned between L479-483, the random effect for stream site should account for variation caused by stream order, distance inland, and elevation that may also drive different species’ habitat use.
- In regard to microhabitat use, please see below comment.

You show no data on the proportional make up of your observations between the species, nor information on equivalence (or not) of size classes between the species. Likewise, there is no exploration of whether there is any spatial or microhabitat separation between the species (e.g. do shortjaws inhabit larger pools than bandeds?). Much of this would be fairly straightforward to illustrate graphically. With respect to species management, these factors are crucial to understand and be confident about for ensuring the validity and credibility of your models for informing management actions.

- Figure 1 now shows the biomass of each species. Because our habitat and fish sampling focused on the 50 m reach scale, we cannot accurately explore microhabitat relationships (e.g., at the pool scale). Attempts to include variables that could potentially be used to examine more microhabitat scales such as average pool volume were unsuitable. For example, reaches could have one large pool and one very small pool, which produced a highly variable average pool size that would not be accurately linked to fish biomass. Nevertheless, current analyses at the reach scale provide practical biomass estimates for each species.

Thirdly, and somewhat linked to the above, how representative are the west coast river systems and kōkopu populations of other rivers and kōkopu populations across New Zealand, and hence, how transferable are the results to elsewhere in the country?

- West Coast river systems and catchments are not notably different from other New Zealand regions that contain kōkopu. Additionally, our standardised predictions should be applicable to kōkopu populations across New Zealand because they describe the realised niche of each size-class and because the random site factor accounts for variation caused by stream order, distance inland, elevation, and disturbance events that often alter kōkopu species’ densities (L479-483).

Fourthly, is there an interaction between pool size and pool depth? Intuitively I would expect the potential for some statistical redundancy between these two variables. It would be good to see this addressed. Likewise, how about between any of the other predictor variables? How closely correlated are they? Similarly, does the apparent correlation between smaller individuals and forest cover simply reflect a preference for smaller streams (and hence greater chance of canopy closure)?

- Pool area and depth have been combined and replaced with pool volume, which better represents available habitat space. As indicated between L206-208, multicollinearity was checked using variance inflation factors and were notably low (<2).
- Unfortunately, the wetted width of the streams were not consistently measured. However, any correlation between wetted width and forest cover would likely be minimised due to the wide viewing angle of the convex densiometer used and because all streams were between 2-6 m wide. Furthermore, we would expect that pool volume and wetted width would be correlated due to larger streams likely having larger pools, but due to low VIF values (<2) between pool volume and forest cover, this indicates that is unlikely that forest cover was correlated with wetted width.

Finally, and this is in my view a particularly critical omission, there is no cross-validation of the statistical models. Good practice in this sort of study would typically see some form of model validation undertaken and reported. For example, using a proportion of the dataset to train the model and a separate portion of the dataset to test and quantify model predictive performance. The addition of this sort of approach would help to provide possible end users greater confidence in the relevance and transferability of the model for real world application.

L226-227: We take your point and did consider it. However, according to Fang (2011), further cross validation analyses are unnecessary because we used maximum likelihood estimates within our AIC analyses that are asymptotically equivalent to the leave-one-cluster-out cross-validation.

More generally, I felt that the introduction was biased towards pelagic, drift-feeding species, without really acknowledging that some of the statements regarding energetic benefits of pool feeding etc may not be as generalisable as could be inferred. You also jump into some fairly bold statements about statistical models in your opening paragraph (e.g. L42) that in my view aren’t as well justified as they could be and perhaps need a bit more introductory context.

Reworded sentence (L48-50) to be less bold. Remaining sentences seem suitable.

Added “drift-feeding” to pool description (L57) because it applies more to drift-feeding fish. However, statements about in-stream cover, forest canopy cover, and size-related hierarchies have been left generally applicable because they apply to most stream fish, whether pelagic or demersal.

One specific comment about your choice of sampling for determining mean pool depth – why only measure depths along one axis of the pool? Can you be sure that is representative of the mean pool depth? Do you have a reference for this method ahead of others (e.g. 10 random depth measurements or 5 on each axis)? I would have thought there was quite a bit of depth variation in the longitudinal axis.

Measurements were taken from the width axis because measurements taken from the length axis were presumed to be biased by following the thalweg, which remained rather consistently deep along the pools. Our method has been noted in the text (L166-167), but we acknowledge this could have been completed more effectively (i.e., take measurements across the width at the beginning, middle, and end of the pool). We do not feel that this will notably alter the accuracy of our pool volume results.

In the discussion (and throughout), I think it would be good to consider your terminology with respect to habitat use/requirements/preference. These three terms have different meanings to me – habitat use being the habitats that fish are using (i.e. where you find them when you look for them), habitat preference being where they would most like to be if the habitat was available, and habitat requirements being habitats that are required to fulfil a particular function. These can all be the same, but could also all be distinct. You talk, for example, about small and large kōkopu having different habitat ‘requirements’ (e.g. L288). Do they really require different habitats? Or do they, as you allude to later, have overlapping fundamental niches but different realised niches (L317-318) due to competition for ‘preferred’ space? Or perhaps there is a genuine spatial segregation of small and large kōkopu habitats, with the distribution of smaller fish skewed towards smaller, lower order streams, and larger fish in larger, main stem streams, that may be more indicative of differing habitat preferences/requirements? I think this could be explored in more depth through your analysis, for example by illustrating the random effects of site to show how these relationships may vary by stream size. I know this may seem somewhat pedantic, but there are crucial distinctions in these terms and what they may mean from a management/restoration perspective, so it’s important to be precise about what you mean.

Terminology has been fixed to reflect our examination of habitat use. As explained in an above comment, stream width was not measured. However, stream related variables like elevation, distance inland, and stream order should be accounted for by the random ‘site’ variable. The variation accounted for by site is now shown in Table 1.
