# Supplementary material for: Predicting biomass of resident kōkopu (Galaxias) populations using local habitat characteristics
Source: PLoS One. 2023 Mar 14;18(3):e0261993. doi: 10.1371/journal.pone.0261993 (PMC10013890; doi:10.1371/journal.pone.0261993)
Supplement: Attachment — Submitted filename: Response to Reviewers.docx [file pone.0261993.s005.docx]

Firstly, in my view it is not appropriate to include the biomass of other species as random effect – this should be treated as a fixed effect because, as you state, it is included to account for the direct effect of competitive exclusion.
Interspecific interactions are likely influential, but we have decided to leave the biomass of other kōkopu species as random variables because our aim here was to examine kōkopu associations with abiotic habitat variables, so we only need to account for biotic influences. Investigating biotic interactions between kōkopu was definitely not an aim and isn’t necessary to predict local kōkopu abundance from habitat features. Furthermore, having the biomass of the other kōkopu species as random variables allows our partial plots to provide the most accurate estimation of each species’ habitat use by accounting for the biomass of other kōkopu present (L227-230). An additional benefit of focusing on abiotic variables is that the models can be used to estimate expected kōkopu biomass from local habitats without spotlighting them, which would otherwise be needed if other species were added as fixed variables in the model (L477-480).

Secondly, it appears that you are treating all sampled reaches as independent sites, even though they are nested within catchments and hence subject to correlated effects. In my view this is potentially an inappropriate treatment of pseudo-replication and the spatial structuring of reaches by catchment needs to be accounted for in your formulation of your site random effect (e.g. reaches need to be nested within catchments).
L220-227: Reaches were used as replicates (for more statistical power estimating habitat-biomass relationships) and a random stream factor (3 reaches within a stream) was added to account for within stream variability. This method provides the strongest statistical inference and accounts for the adjacency of reaches. Including a random catchment factor is unnecessary because there were few cases where streams were found in the same catchment, and would lead to further analytical problems due to uneven replication among catchments. Finally, we have already accounted for key factors that would likely cause catchment variation by ensuring there were no downstream barriers, all reaches and streams were accessible to each species of kōkopu, and selecting catchments that were minimally impacted.

Thirdly, I am not yet sufficiently convinced of the transferability of your models to areas beyond the geographical and environmental space within which the data have been collected. The west coast rivers of New Zealand have a fairly unique character – short, steep rivers, high rainfall, glacial derived flows, relatively intact catchments (at least compared to a lot of New Zealand). I think that compared to places like the Waituna (see Greer et al 2012) or central Waikato (see Franklin et al 2015) strongholds for giant kokopu, or Coromandel (see West et al 2005) or Auckland (see McEwan et al 2009) strongholds for banded kokopu, there are substantial differences in the stream type, flow regimes, and habitat structure where these species are resident. Similarly, there are relatively few places elsewhere in New Zealand where all three kokopu species co-occur in the same way (see Yungnickel 2017). These models may represent the realised niche in this region, but given the different habitat structures and relative differences in species composition and dominance in other regions, I don’t think you provide sufficient evidence to be able to claim that the realised niche elsewhere would be equivalent and thus that your models are transferable. I would like to see some evidence to support this claim – for example how do the environmental gradients you have sampled compare to other regions? While it does not cover extrapolation of mixed effects models, the paper by Booker & Whitehead (2018) may be of interest in this respect.
Our models estimate biomass from minimally impacted catchments with no migratory impediments. That is entirely appropriate since the goal of our study was to evaluate ‘local’ influences and other landscape scale influences on distribution would confound our patterns. We have strengthened the Introduction, Methods and Discussion text to further clarify that point and acknowledge the need to account for other landscape-scale drivers (e.g., L97-99, L153; L472-474; L480-484). Furthermore, the Conclusion emphasises that these estimations will thus provide a baseline that should indicate if kōkopu biomass is influenced by other environmental influences (i.e., a barrier; L494-496). These estimations should not be biased by our site selection because they were not glacially derived, abnormally steep or elevated (>100 m elevation), and spanned up to 17km inland (to ensure we did not exclude giant kōkopu; L151-153). Nevertheless, we now acknowledge in the manuscript that long-term widespread monitoring is required to confirm our patterns (488-490). Although the West Coast does have high rainfall, we do not believe this would notably influence our local habitat results because tagged kōkopu in these streams take refuge during the floods and typically remain within the same reaches (unpublished data). Finally, constructing a model like the one being asked for requires repeating a study like this in different areas so the large-scale influences were properly incorporated. Such an approach would also need to encompass the range of landscape-scale influences (e.g., by incorporating habitats close to and far from the sea). We clearly do not have the data to do. Moreover, given realistic resourcing constraints, we have actually done the best thing to produce the most applicable local model by working in least impacted catchments and thereby controlling for most other influences that would confound local habitat use.

Regarding species overlap, Yungnickel’s (2017) Figure 2.1 (assuming that is the figure being referenced) shows considerable kōkopu distribution overlap, particularly across the north and west coast of both main islands. However, as long as the models are applied within the targeted kōkopu species’ natural range, they should provide an accurate standardised estimation (regardless of which other kōkopu species are present) due to incorporating reaches that included a variety of kōkopu co-occurrence combinations. We now mention this in the text and provide a Table in supplementary materials that describes the range of different assemblages our data encompass (S1 Table; L474-484). Moreover, including the density of other kōkopu as a random effect in the models parses this variance, thereby providing generally applicable local models.

Fourthly, your site random effect accounts for around half of the variation in biomass in your models of both banded and giant kokopu. Given the general spatial structuring of kokopu species with distance inland and elevation, this is not surprising. However, it again raises the question of transferability of your models to other sites. Why not reduce some of this random error tied to site by explicitly accounting for distance inland/elevation (I appreciate there’s some correlation there!) and potentially other landscape scale variables as fixed effects in a way that will make the results more transferable? Similar approaches using landscape scale predictors have been used to predict fish probability of occurrence across the New Zealand river network previously (e.g. Leathwick et al 2005), so it seems logical that a similar approach here to cover off the broader biogeographical drivers before accounting for local scale variations in biomass associated with the site level variables should be explored.
This argument is already mostly covered above. We agree that large scale factors are important to consider and certainly account for large amounts of variation in some places, but we focus on local habitat variables in least impacted catchments to provide the best possible model of local habitat conditions. Our sites didn’t cover great distances inland (max 17 km), elevations (<100m), and were not abnormally steep, so those influences have been controlled for. As already described, we now highlight that our study is focused on evaluating kokopu abundance at a local scale and is not about predicting the spatial distribution of kokopu across the landscape. Thus, further confusion about the aims should be avoided. Moreover, in the absence of a mega kōkopu study involving collection of local habitat use across many regions, our models provide the *best* and *most applicable* evaluations of local habitat use because we have controlled for the landscape-scale influences the reviewer is concerned about. For example, if a local manager anywhere in the country wants to construct the best local kōkopu habitat, they can use our results (the landscape features will control whether kōkopu get there, but they’ll still have appropriate habitat). Finally, we’re not going to change the paper’s aim away from local influences because our data do not provide any strength of inference to assess those (i.e., they were collected in least impacted catchments close to the coast and at low altitude, so there in no ability to assess those effects).

It is my view that these issues of model structure and transferability need to be better addressed to strengthen this manuscript and improve the usefulness of the models. A few more specific comments follow:

1. I remain unconvinced that your surveys coinciding with spawning season for these species could not have impacted their distribution/behaviour. The Charteris et al manuscript you cite as a justification that kokopu spawn in situ explicitly states that the giant kokopu from that study site migrated out of the reach and then returned following spawning. Similar observations have been observed for giant kokopu by David & Closs 2002. I appreciate that the Franklin et al 2015 paper showed giant kokopu spawned in situ, but this reflects the current level of uncertainty regarding spawning behaviour in spawning kokopu – it’s rarely been documented comprehensively but there’s enough evidence to suggest both aggregation of fish within areas and movement between reaches for spawning.
L144-147: We know of only one source suggesting that ripe giant kōkopu migrate downstream in McDowall (1990); there was no further information or source for these observations, so they are speculative. McDowall (1990) also states that ripe and spent banded and giant kōkopu are found in typical adult habitats and will likely not migrate far to spawn. Additionally, Charteris (2002) found that shortjaw and other galaxiid species generally stayed within their resident home ranges over the spawning season. When fish did move out of their home ranges, it was not thought that it was a direct result of spawning, as it was not limited to spawning time. Thus, we don’t think the movement argument stacks up. Furthermore, we did not find fish in a condition that suggested they had recently spawned or were about to spawn.

Charteris, S. C. (2002). *Spawning, egg development and recruitment of diadromous galaxiids in Taranaki, New Zealand.* MSc. thesis, Massey University, Palmerston North, New Zealand.

McDowall, R. M. (1990). *New Zealand freshwater fishes: a natural history and guide*: Auckland, Heinemann Reed.

2. Some previous studies of giant kokopu have demonstrated clear seasonal variation in activity and microhabitat use (e.g. David & Closs 2003). Please address this in your discussion – what does the timing of your surveys mean for the applicability of these models?
L484-488: We now acknowledge that patterns may vary seasonally due to small kōkopu immigration events in spring, and the subsequent gain and loss of large kokopu biomass associated with spawning in autumn. However, it is unlikely that seasonal shifts in kōkopu microhabitat use (i.e., position within a pool) will influence our local habitat associations substantially because fish generally remain within the same relatively short home range (David & Closs, 2003).

3. In all of your tables I think you mistakenly use BK instead of BC to indicate your bank cover fixed effect. If this is not a mistake, I suggest using a different abbreviation for bank cover given that BK is also used to mean banded kokopu in the paper.
This was a mistake and has now been fixed.

4. In your discussion I think it would be helpful to consider what additional value your models have over existing models of fish distribution available for New Zealand (e.g. Leathwick et al 2005). Please compare and contrast the relative strengths and weaknesses of these approaches and, therefore, the suitable contexts for their application.
L469-480: The advantages of our localised models over pre-existing New Zealand fish distribution models is that they (1) provide standardised biomass estimations for small and large size-classes instead of species presence; (2) were developed using consistent methodology at an explicitly local spatial scale; and (3) avoided confounding influences by only included streams with no downstream barriers, invasive species, or notable degradation. Furthermore, these standardised predictions are applicable to kōkopu populations across New Zealand, regardless of which other kōkopu species are present, because they account for the influence of other kōkopu species and were developed using reaches that included most combinations of kōkopu co-occurrence (S1 Table). By accounting for biotic interactions between kōkopu species in model development, more accurate standardised biomass estimations can be made using local habitat features without needing to spotlight stream reaches.
L483-484: We have noted the limitations of our models, in that they (1) can only be applied to reaches that are within the natural distribution of the target kōkopu species, and (2) may vary regionally, particularly when applied to areas less accessible to kōkopu or with more impacted catchments. However, as stated above and throughout the manuscript, the models will work most effectively when other factors are accounted for. Furthermore, they are ideal for identifying the best local habitat for managers to construct anywhere (as described above).
