# Supplementary material for: Predicting biomass of resident kōkopu (Galaxias) populations using local habitat characteristics
Source: PLoS One. 2023 Mar 14;18(3):e0261993. doi: 10.1371/journal.pone.0261993 (PMC10013890; doi:10.1371/journal.pone.0261993)
Supplement: Attachment — Submitted filename: Response to Reviewers.docx [file pone.0261993.s006.docx]

Reviewer #2: Dear Authors,

Reviewer #2: It is great to see the ongoing improvements to this manuscript in response to prior review comments. It retains its strong empirical foundation and in acknowledging and addressing some of the limitations around transferability and application relative to other species distribution modelling approaches I think now gives a more balanced view of its applied value. However, I believe you have not appropriately addressed the errors in the structure of your statistical models as highlighted by previous reviews.

Reviewer #2: The structure of the model (in terms of what is included as fixed v random effects) should not be determined by convenience to support a desired narrative. It must be dictated by the protocols of the statistical methodology. In that respect, you must include the biomass of other species as a fixed effect. It is a continuous variable, the value of which has real meaning, and as such can only be treated as a fixed effect. My understanding is that random effects must be factors and that the levels of those factors should effectively be ‘meaningless’ in so far that you can use any set of labels and it doesn’t change the interpretation of what it means (e.g. labelling sites 1, 2, 3 versus A, B, C). As such, I’m not even sure how you’ve included biomass as a random variable as it presumably would have had to be converted to a factor somehow (maybe high/medium/low?), but even if you’ve done this the factor labels have meaning – they’re ordered – and so do not meet the normal rules of defining a random effect. I appreciate that this may appear ‘inconvenient’ for how you are wanting to frame your narrative, but assuming that biomass is retained as a significant fixed effect in your models, then I think it can potentially help with the transferability of the models – it would allow someone who is trying to apply the model in the real world to set biomass of a species at zero, if for example, it was known not to co-exist at the site. Similarly, you could provide median and/or confidence intervals around your observed biomass values that someone wishing to use the model could utilise to get an understanding of the range of biomass in the target species given the local habitat conditions. My key point is that just by including biomass of competing species as a fixed effect, it doesn’t negate the value of the model – in fact I would argue it strengthens the potential utility because it allows users to more appropriately recognise uncertainty.

Authors: L224-230: We originally included the biomass of other kōkopu size classes as fixed effects to test for potentially confounding interspecific interactions, but these were removed because we found no associations between kōkopu of the same size class (S1 Fig, S1 Table). Furthermore, any direct effect of kōkopu biomass on the response, particularly between size classes, would likely be indirectly driven by abiotic variables, and we are unable to effectively disentangle abiotic and biotic effects. To effectively isolate biotic and abiotic effects, we would need to include interactions between each kōkopu species’ size class and each habitat variable, which would require us to not only cover a wide array of different abiotic conditions, but also replicate this abiotic array with different kōkopu co-occurrences, which would require a naturally unrealistic dataset that we do not possess. Moreover, including the biomass of other size classes as an additive effect would lead to misleading habitat associations because of the confounding effects between abiotic and biotic predictors (as stated above). Therefore, we have not included biotic variables in our models at all.


***Added because this is relevant to early review comments about kōkopu grouping***
Authors: L208-212: We have combined the biomass of the small kōkopu size classes because juvenile shortjaw and giant kōkopu were absent from most reaches due to being naturally rare, which meant we could not develop effective habitat-biomass models for these size classes. However, this grouping should not confound our results because juvenile kōkopu likely occupy the same habitats due to being competitively displaced by larger dominant congeners. We found that our partial plots and models became slightly more accurate because of this grouping, indicating that the small number of juvenile giant and shortjaw kōkopu present were likely using similar habitats to small banded kōkopu.

Reviewer #2: Another component of your GLMM that you need to address is the treatment of site/reach. Your use of the term ‘site’ as the term in the model appears to have created some confusion; to most people at first glance this would be interpreted as the lowest level of replication, but I think in this case this is what you term a ‘reach’. The prior review comments remain valid in this respect in so far that it would be most appropriate for ‘reach’ to be nested within ‘site’ within your model formulation. This will appropriately address the spatial structuring of your data and currently inappropriate treatment of pseudo-replication.

Authors: We believe the manuscript adequately explains our spatial structuring and used terms. We firstly introduce our spatial structuring in the beginning of our methods:
“To investigate which habitat features are most strongly associated with reach-scale kōkopu biomass, three 50-m reaches were sampled within each of 19 streams…” (L144-145).
Then in our analytical methods, we introduce and explain the ‘site’ term and our site/reach spatial structuring in an analytical context:
“Linear mixed-effects models were constructed using the ‘lmer’ function and included a random factor for stream site, hereafter referred to as ‘site’. A random factor for site was included so that each of the three reaches nested within each site could be used independently to examine how habitat characteristics influenced kōkopu biomass.” (L231-236).

Authors: Because each reach was not sampled more than once within each stream, and the three reaches sampled per stream are the only pseudo-replicates we have for each model, only a random variable for site is needed. A random variable for reach nested within site does not change our results, because there is no variability within each reach (one sample per reach). A random effect for reach could only be included if we used one global model that tested for habitat associations between classes and species, meaning each measurement of biomass for each species and class would be a pseudo-replicate. However, because we have independent models for each size class of each species, we cannot do this. We created this global model to test if a random reach effect was influential, but it explained 0% of our random/error variation. We have chosen to stick with independent models because 1) we can obtain an R^2^ for each size class, 2) the partial plots for independent models are far more informative, and 3) because we can test for relative variable importance for each case.

Reviewer #2: More generally, I feel that the comments made in the previous review around the uncertainty of the potential impacts of spawning remain valid – I appreciate your responses, but you rightly acknowledge that there remains considerable uncertainty over the role of migratory movements around spawning. As such, I feel that you could provide a more balanced acknowledgement of this in your manuscript. It’s not a fundamental flaw, but there appears to be enough evidence to suggest that particularly for giant kokopu, at least in some streams, they likely undertake spawning migrations and at the moment you largely dismiss this possibility – just be honest about the uncertainty.

Authors: L146-151: We have now emphasised in the manuscript that there is uncertainty around kōkopu spawning behaviours and giant kōkopu spawning migrations. But as previously stated, we cannot find any evidence to suggest kōkopu migrate for spawning, whereas our cited studies state that all three species of kōkopu generally remained within their home range or did not move far to spawn. If you have a link to any of this evidence we will readily include it.

Reviewer #2: I feel that there is a strong manuscript here, but please don’t ignore good statistical practice for the sake of fulfilling a particular narrative. Likewise, it is always advisable to provide a balanced and honest discussion of the limitations/uncertainty in your work – this improves its credibility and is not something to be afraid of.
Best of luck with completing your manuscript.
